# Green-Synthesized Copper and Copper Oxide Nanoparticles: Structural Characterization and Evaluation of Biological Activity

**DOI:** 10.3390/antiox15030339

**Published:** 2026-03-07

**Authors:** Ionut Iulian Lungu, Alina Stefanache, Nicoleta Anton, Andreea Lungu, Vera-Maria Platon, Andreea-Maria Mitran, Oana Cioanca, Cornelia Mircea, Monica Hancianu

**Affiliations:** 1 Grigore T. Popa University of Medicine and Pharmacy Iasi, 700115 Iasi, Romania; ionut-iulian.lungu@umfiasi.ro (I.I.L.); anton.nicoleta1@umfiasi.ro (N.A.); andreea-lungu@umfiasi.ro (A.L.); andreea-maria.mitran@umfiasi.ro (A.-M.M.); cornelia.mircea@umfiasi.ro (C.M.); monica.hancianu@umfiasi.ro (M.H.); 2 Ophthalmology Clinic, Sf. Spiridon Emergency Clinical Hospital, 700111 Iasi, Romania; 3 Institute of Gastroenterology and Hepatology, “St. Spiridon” Emergency County Hospital, 700111 Iasi, Romania; 4 Petru Poni Institute of Macromolecular Chemistry, 700487 Iasi, Romania; platon.vera@icmpp.ro

**Keywords:** copper nanoparticles, green synthesis, capping agents, sustainable nanomaterials

## Abstract

Copper-based nanoparticles, especially metallic copper (Cu NPs) and copper oxide (CuO NPs), have attracted increasing attention due to their redox activity, biological efficacy, and technological applications. However, conventional chemical synthesis often involves toxic reagents, limiting their biomedical applicability. In this context, plant-mediated green synthesis has evolved and has become a sustainable and cost-effective alternative. This review provides a comprehensive overview of recent advances in the biosynthesis of Cu and CuO nanoparticles using plant extracts. The main synthesis pathways are examined, with emphasis on the role of phytochemicals as reducing, stabilizing, and capping agents, as well as the influence of reaction parameters on nanoparticle yield. The review highlights the diversity of plant species and extract types used and clarifies their effects on nanoparticle size, morphology, oxidation state, and surface chemistry. Key physicochemical characterization techniques (ultraviolet–visible (UV–Vis) spectroscopy, Fourier transform infrared (FTIR) spectroscopy, X-ray diffraction (XRD), electron microscopy, and zeta potential analysis) are systematically discussed. Moreover, a summary of in vitro and in vivo biological activities is provided, including antimicrobial, antioxidant, cytotoxic, anticancer, wound-healing, and plant-growth-promoting effects. Overall, plant-mediated copper-based nanoparticles demonstrate significant potential as biofunctional nanomaterials. Nevertheless, challenges concerning reproducibility, mechanistic understanding, standardization and toxicological evaluation must be addressed to facilitate reliable translation into biomedical applications.

## 1. Introduction

Copper nanoparticles (Cu NPs) and copper oxide nanoparticles (CuO NPs) are among the most versatile nanomaterials in modern nanoscience. Their growing interest is linked to unique physicochemical properties, significant biological activity, and a wide range of technological applications [1,2,3,4]. Copper’s electronic configuration allows it to alternate between Cu^0^, Cu^+^, and Cu^2+^ oxidation states, which enables redox activity. This characteristic is essential for catalytic processes, free radical production and antimicrobial behavior [5,6,7].

As particle size decreases into the nanoscale domain, the surface-to-volume ratio increases dramatically and enhances interactions with electron-rich compounds and biological molecules. As a result, both catalytic efficiency and biological reactivity are significantly improved. Compared with widely used noble metal nanoparticles such as silver and gold, copper offers greater abundance, lower cost, and suitability for industrial-scale applications [8,9,10].

Traditional chemical synthesis of Cu-based nanoparticles usually involves strong reducing agents, organic solvents, stabilizers, and energy-intensive processes. Although these methods allow good control over particle size and crystallinity, they raise environmental, toxicological, and economic concerns [11]. Harsh reaction conditions can lead to toxic residues, hazardous waste, or nanoparticles with unwanted surface contaminants. Such issues limit their suitability, particularly for biomedical applications. As a result, research has increasingly focused on alternative synthesis methods that minimize the use of hazardous chemicals and reduce ecological impact [12,13].

Among green synthesis approaches, such as methods based on bacteria [14], fungi [15] and algae [16], plant-mediated synthesis has gained particular attention [17]. This type of method is valued for its simplicity, good reproducibility, low energy consumption, and the wide availability of plants [18]. Plant extracts are rich in secondary metabolites such as flavonoids, terpenoids, tannins, phenolic acids, organic acids, sugars, alkaloids, and proteins. These compounds can simultaneously act as reducing, stabilizing, and capping agents during nanoparticle formation. Their chemical diversity enables the reduction of copper ions to metallic or oxide forms and supports the stabilization of the resulting nanostructures. In addition, they can influence particle morphology and surface chemistry. As a result, this eco-friendly method often produces nanoparticles coated with bioactive plant-derived compounds, which may enhance antimicrobial, antioxidant, anticancer, and anti-inflammatory properties [19,20,21].

Plant-mediated synthesis of Cu and CuO nanoparticles has grown dramatically in the past ten years [12,22,23,24]. Numerous plant species, such as medicinal herbs, shrubs, fruits, roots, leaves, peels, barks, seaweeds, and even agricultural waste, have been studied; each has unique metabolic profiles that affect the formation of nanoparticles [17]. In this article, we present a comprehensive overview of plant-based research on copper nanoparticles, including original experiments, mechanistic insights, and biological evaluations. Moreover, it supports a critical comparison of results across plant extracts and synthesis mechanisms.

Despite the rapid growth of research in this field, several methodological challenges remain. Plant extracts are inherently complex and vary widely depending on species, cultivar, growth conditions, season, and extraction methods. As a result, reported nanoparticles often differ significantly in yield, size, morphology, and biological activity, which limits cross-comparison and standardization between studies. Moreover, many reports focus mainly on preliminary evaluations, particularly antibacterial assays, without in-depth mechanistic analysis or broader biological assessment. Toxicological studies, although widely acknowledged as essential, are still insufficiently addressed in many publications. This review focuses on these challenges by identifying recurring patterns and research gaps within a broader scientific context.

The review is organized around four main themes. First, it discusses biosynthesis pathways, with emphasis on phytochemical involvement, reaction conditions, plant species diversity, and mechanistic aspects. Second, it integrates findings from common characterization techniques, highlighting the role of UV–Vis spectroscopy, electron microscopy, FTIR, and XRD in confirming nanoparticle formation and structure. Third, it reviews in vitro biological studies, including antimicrobial, antifungal, antioxidant, cytotoxic, anticancer, and catalytic activities. Finally, the review covers in vivo studies that provide the highest level of biological validation. These include wound-healing, antileishmanial, neuroprotective, plant-growth-promoting, and toxicological studies. The review ends with a critical overview, identifying strengths, challenges, and future research needs.

## 2. Biosynthesis of Copper Nanoparticles Using Plant Extracts

**Figure 1:**
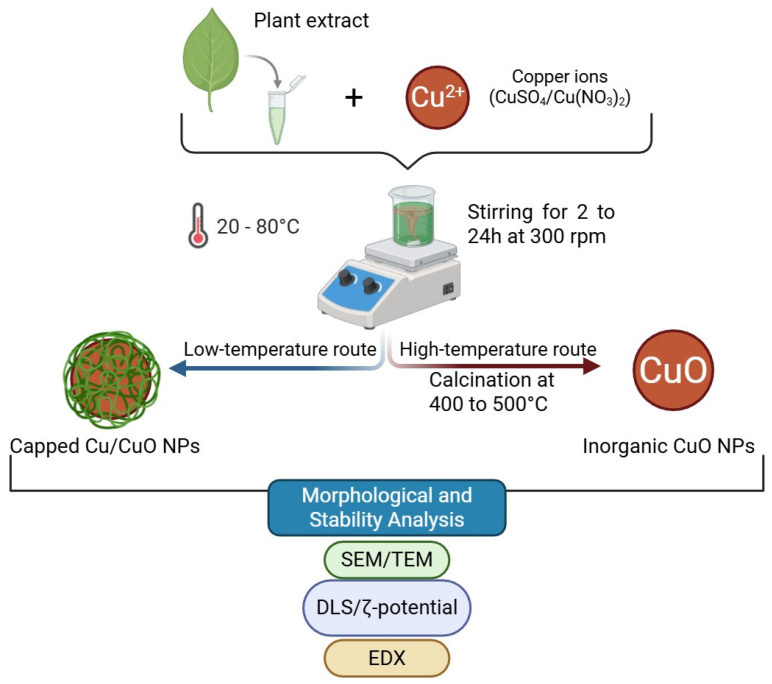
Green-synthesis of copper and copper oxide nanoparticles. Created in BioRender. Lungu, I. I. (2026).

Plant-mediated biosynthesis of Cu and CuO nanoparticles typically involves the reaction of copper salts with aqueous or hydroalcoholic plant extracts. Commonly used copper precursors include copper sulfate [25], copper nitrate [26,27], and copper chloride [12]. Most techniques involve boiling, heating, or extracting the plant material at controlled temperatures to produce solutions rich in polyphenols, flavonoids, reducing sugars, ascorbic acid and proteins [22,28]. When these extracts are mixed with copper salts, the biomolecules reduce copper ions and simultaneously stabilize the newly formed nanoparticles by complexation. This process is illustrated in Figure 1. Overall, green synthesis can be considered a simple “one-pot” method, in which reduction, stabilization, and capping occur in a single step.

### 2.1. Role of Phytochemicals in Reduction and Stabilization

Phytochemicals play a key role in forming nanoparticles. Phenolic compounds are especially important because their hydroxyl groups can easily donate electrons to Cu^2+^ ions, reducing them to Cu^0^. In this process, phenolics oxidize to quinones, which may also help stabilize the nanoparticles through π–π interactions or covalent bonds [29]. Because flavonoids have multiple hydroxyl groups that can participate in redox reactions, they function similarly. Other reducing agents include ascorbic acid, sugars, and terpenoids. Through amino, hydroxyl, or carbonyl groups, compounds such as proteins, polysaccharides, and alkaloids bind to the surfaces of nanoparticles and provide steric stabilization.

Studies using extracts obtained from *Blumea balsamifera* [30], *Moringa oleifera* [31], and *Jatropha curcas* [32] show that flavonoids and phenolic acids are important for both reducing copper ions and capping the nanoparticles. Plant extracts rich in terpenoids, such as those derived from *Piper retrofractum* [33], exhibit fast reaction kinetics, which corresponds to the strong reducing power of these compounds. Root extracts from *Krameria* spp. [34], containing tannins and phenolic lipids, produce nanoparticles that remain stable in solution for long periods. This highlights the role of capping molecules in keeping nanoparticles well-dispersed.

### 2.2. Diversity of Plant Species and Extract Types

The plant species used in the evaluated studies are very diverse and includes medicinal herbs, edible plants, agricultural waste, and non-edible species. This variety brings complex phytochemical profiles and different mechanisms of nanoparticle formation. Extracts from plants like *Withania somnifera* [35], *Hyptis suaveolens* [36], *Thymus fedtschenkoi* [37], *Ephedra alata* [38], *Achillea biebersteinii* [39], *Rosmarinus officinalis* [40,41], *Mentha species* [42,43,44,45], *Matricaria chamomilla* [46], and *Cinnamomum* species [47] have strong bioactive compounds that are also used in traditional medicine. These substances are effective as both reducing and capping agents, and often help to produce nanoparticles with good biological activity.

Many studies also focus on fruit-based extracts, such as those from strawberry [48], *Citrus sinensis* [49], *Citrus paradisi* [50], and *Morinda citrifolia* [51]. *Citrus* fruits, in particular, contain ascorbic acid, citric acid, flavonoids, and limonoids, making them strong reducing agents. Fruit peels and wastes, like those from *Citrus reticulata* [52] and hawthorn berries [53], are popular in green synthesis because they are cheap, easy to find, and rich in phenolic compounds.

Leaf extracts from plants such as *Ageratum houstonianum* [54], *Hagenia abyssinica* [55], *Catha edulis* [56], *Avicennia marina* [57], and *Malva sylvestris* [58] are also commonly used. They have high levels of chlorophyll, flavonoids, and polyphenols, which give them strong reducing and stabilizing abilities. These extracts often produce nanoparticles with uniform size distribution.

Non-edible plants and waste materials have also shown good results. For example, extracts from *Vaccinium* waste [59], tamarind shell powder [60], and gum Arabic [61] shows that agricultural byproducts are useful in nanoparticle synthesis.

### 2.3. Influence of Reaction Conditions

**Figure 2:**
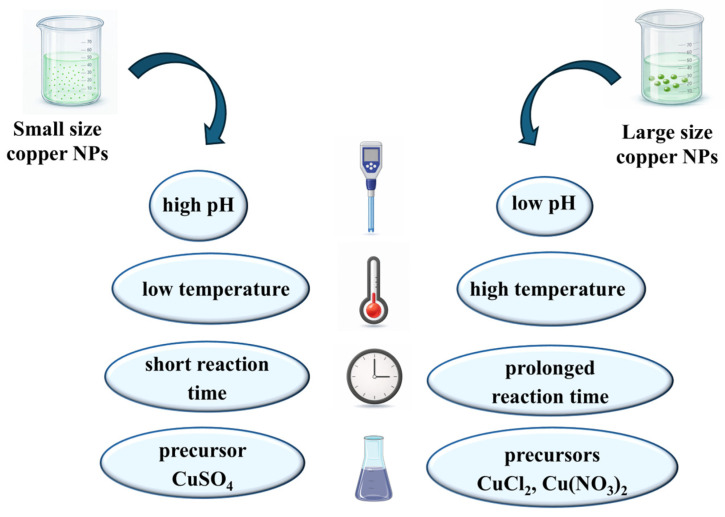
Effect of synthesis parameters on copper nanoparticle size. Created in BioRender. Lungu, I. I. (2026).

Nanoparticle properties depend on reaction factors such as pH, temperature, reaction time, plant extract and concentration of the precursor. Although numerical data were not provided in the reference text, general patterns were found throughout the literature (Figure 2).

#### 2.3.1. pH and Temperature

Most studies report that increasing pH facilitates the deprotonation of phenolic hydroxyl groups, thereby enhancing their electron-donating capacity. Moreover, higher pH values typically yield smaller, more uniform nanoparticles with improved stability. Similarly, higher temperature increases reaction kinetics and nucleation rates. Micro-wave-assisted synthesis, as demonstrated in the study using *Citrus sinensis* extract [49], substantially reduces reaction time by enabling rapid molecular heating and uniform energy distribution. Furthermore, the reaction temperature exerts a significant influence on nanoparticle dimensions [62]. Usually when reaction temperature increases, larger CuO nanoparticles are formed. Thus, particle sizes can increase from 9–11 nm at lower temperature to 19–45 nm at higher temperature [63].

#### 2.3.2. Influence of Reaction Time and Storage Stability

Reaction time represents a critical kinetic parameter during green synthesis, directly influencing nucleation, growth, and aggregation processes. Shorter reaction times generally favor the formation of smaller nanoparticles due to limited crystal growth, whereas prolonged reaction periods may promote particle enlargement or aggregation [64,65,66]. For example, in the green synthesis using *Ailanthus altissima*, a reaction time of 4 h resulted in smaller nanoparticles (5–20 nm), while extending the reaction to 16 h in the *Allium eriophyllum* system led to larger particles (30–35 nm) [64,67]. These findings highlight the time-dependent nature of nanoparticle growth dynamics.

In addition to reaction kinetics, nanoparticle stability over time (shelf life) is an important post-synthesis parameter that determines long-term performance. During storage, nanoparticles may undergo aggregation, surface oxidation, or gradual structural modification, particularly in systems lacking sufficient phytochemical capping. Colloidal stability is closely related to surface charge, organic coating integrity, and environmental conditions. Therefore, temporal stability must be considered separately from synthesis kinetics, as it directly impacts reproducibility and practical applications of green-synthesized copper-based nanomaterials.

#### 2.3.3. Plant Extract and Precursor Concentration

The concentration of plant extract is important because it acts as both a reducing and stabilizing (capping) agent in the production of CuO nanoparticles [28]. Berra and colleagues showed that using a higher amount of plant extract increases the rate of copper-ion reduction; this leads to the formation of more Cu and CuO nanoparticles. This happens because the plant active metabolites are responsible for both reducing the copper ions and keeping the nanoparticles stable [68].

The type of copper salt used as a precursor also affects the crystallinity, oxidation state, and shape of the nanoparticles. For example, a study using *Malva sylvestris* extract compared copper sulfate, copper nitrate, and copper acetate as precursors and found that the resulting nanoparticles had different characteristics. The size of the CuO nanoparticles varied between 19 and 26 nm, the smallest being obtained with copper sulfate. Nanoparticles synthesized from copper nitrate and copper chloride showed a higher degree of crystallinity [58]. This observation highlights the importance of precursor selection for designing nanoparticles with specific physicochemical properties.

### 2.4. Formation of Metallic vs. Oxide Nanoparticles

Under normal conditions, especially when exposed to air, copper nanoparticles tend to oxidize easily, forming CuO or Cu_2_O. As a result, many studies report copper oxide nanoparticles as the main product, with no detectable metallic copper, particularly when longer reaction times or higher temperatures are used. For example, synthesis with *Ephedra alata* extract mainly produced CuO nanoparticles [38], while *Morinda citrifolia* extract led to CuO nanostructures with a higher crystallinity [51]. The formation of oxide nanoparticles is often associated with improved photocatalytic and antimicrobial activity, because of higher surface reactivity and the presence of oxygen vacancies [4,69].

## 3. Physicochemical Characterization and Confirmation of Nanoparticle Formation

**Figure 3:**
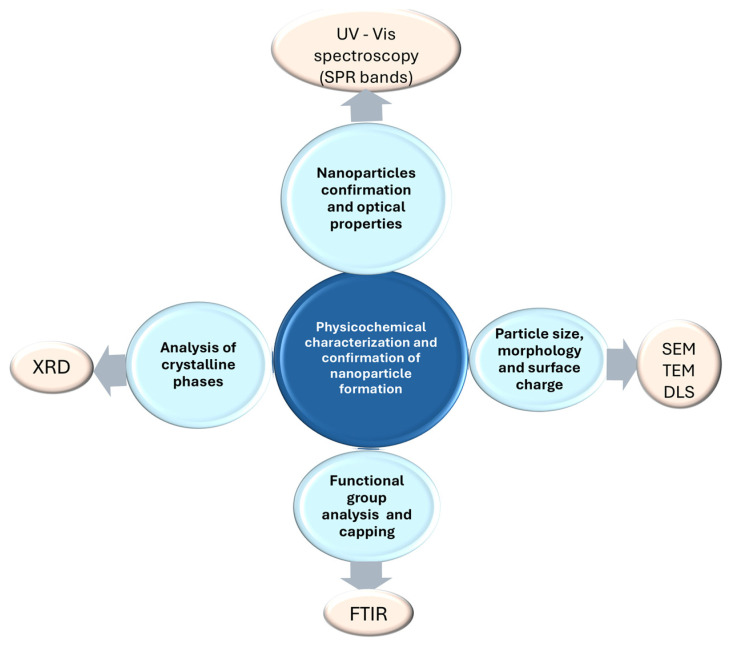
Physicochemical characterization of copper-based nanoparticles.

Literature shows that the formation of copper-based nanoparticles is commonly confirmed using several techniques that are complementary (Figure 3), including UV-Vis spectroscopy, FTIR, XRD, electron microscopy (scanning electron microscopy (SEM)/transmission electron microscopy (TEM)), and dynamic light scattering or zeta potential analysis [70,71,72,73]. In plant-mediated synthesis, nanoparticles typically show a relatively narrow size range, usually between 5–100 nm, with spherical or quasi-spherical shapes. These characteristics are influenced by the reducing strength of the plant extracts and can conduct to different biological activity of the resulting nanomaterials [74].

### 3.1. UV–Vis Spectroscopy and Surface Plasmon Resonance

UV-Vis spectroscopy is usually the first technique used to confirm nanoparticle formation [73,75]. In green synthesis using *Krameria* sp. root extract, Alshammari and colleagues observed a surface plasmon resonance (SPR) band between 406 and 410 nm during reaction optimization. The strongest and sharpest peak appeared at 426 nm when 0.3 M CuSO_4_ was used, indicating the highest yield of copper nanoparticle formation [34]. The narrow shape of this band suggests the presence of a uniform population of metallic Cu^0^ nanoparticles.

In a Heliyon study using seedless date extract, authors reported a single SPR band at 576 nm for Cu/Cu_2_O nanoparticles [71]. This shift toward longer wavelengths compared with the *Krameria* system is related to a larger average particle size, around 78 nm as measured bydynamic light scattering (DLS). It also reflects the mixed Cu/Cu_2_O composition and possibly a thicker organic coating created by the extract. In both cases, the presence of a single, well-defined band in the visible region supports the formation of nanoscale copper materials rather than bulk copper.

Copper oxide nanoparticles often show SPR bands at shorter wavelengths, sometimes in the UV region. For example, in the synthesis of CuO nanoparticles using *Morinda citrifolia* leaf extract, Priya and co-authors reported a clear absorption band at 256 nm, which was assigned to CuO nanoparticles [51]. The gradual increase in absorbance at this wavelength during the first two hours was used to monitor reaction progress. This increase occurred alongside a visible color change from pale green to dark brown, reflecting the continuous formation and growth of CuO nanocrystallites.

Overall, plant-mediated synthesis can generate copper-based nanostructures exhibiting absorption features across a broad spectral interval (≈250–600 nm). However, these features do not originate from a single plasmonic mechanism. The visible bands (≈400–600 nm) are associated with the localized surface plasmon resonance (LSPR) of metallic Cu domains, whereas the UV absorption (≈250–350 nm) is attributed to interband or band-gap related transitions of Cu_2_O/CuO phases. Therefore, the broad spectral response reflects phase heterogeneity (metallic and oxidized copper species) rather than size effects alone.

### 3.2. XRD Confirmation of Crystalline Phases

XRD analysis is commonly used to confirm the crystalline structure and phase composition of copper-based nanoparticles [76,77]. In a synthesis using date extract, Cu/Cu_2_O nanoparticles showed diffraction peaks at values of 43.27°, 50.41°, and 74.17°. These peaks correspond to the (111), (200), and (220) planes of face-centered cubic metallic copper, and additional signals attributed to Cu_2_O [71]. The presence of both metallic and oxide phases is often observed when phenolic-rich extracts partially reduce Cu^2+^ to Cu^0^, followed by surface oxidation when exposed to air.

Similarly, Alshammari et al. reported that nanoparticles synthesized with *Krameria* root extract displayed characteristic fcc copper reflections at the (111), (200), and (220) planes [34]. Selected area electron diffraction (SAED) patterns confirmed their crystalline nature and their distinctive planes. Based on Scherrer equation, crystallite sizes were estimated to be in accordance with TEM measurements, indicating particle sizes of 5–8 nm.

In contrast, CuO nanoparticles synthesized using *Morinda citrifolia* leaf extract showed XRD patterns characteristic of monoclinic CuO, with no detectable secondary phases or impurities. The medium calculated crystallite size was 29 nm, which is close to particle sizes observed using SEM and TEM. This agreement between XRD and microscopy is important when we correlate structural features to biological performance. For example, modest zones of inhibition can be explained by larger particle size and the presence of oxidized forms compared with smaller metallic Cu nanoparticles [51].

More complex systems, including fucoidan-coated CuO or CuS nanostructures used for anticancer or photothermal applications, also rely on XRD to confirm both the copper phase and the presence of crystalline coatings or hybrid structures [78]. Structural features identified using XRD, SEM and TEM can be further connected to the therapeutic efficacy of nanoparticles.

### 3.3. Particle Size, Morphology and Surface Charge

Images obtained with TEM and SEM are commonly used to determine nanoparticles size and morphology [79,80,81]. In case of *Krameria*-derived Cu nanoparticles, TEM analysis showed spherical to slightly oval particles, with sizes between 5.2–7.7 nm [34]. The very small particle size is important, as these nanoparticles produced in vitro large inhibition zones (up to 44 mm). Such behavior is sustained by the high surface-area-to-volume ratio, which allows more effective interaction with microbial membrane.

TEM analysis of Cu/Cu_2_O nanoparticles obtained with seedless date extract revealed the presence of spherical nanoparticles. DLS measurements showed a mean hydrodynamic diameter of 78 nm and a zeta potential of +41 mV [71]. Although the particle diameter was relatively large, the high positive zeta potential suggests good colloidal stability and indicates that can interact electrostatically with negatively charged bacterial membranes.

CuO nanoparticles synthesized using *Morinda citrifolia* extract showed mostly spherical particles with a polydisperse size distribution between 20 and 50 nm, as observed by TEM. SEM analysis indicated an average particle size of 29.2 nm [51]. Energy-dispersive X-ray spectroscopy (EDX)results showed an elemental composition of approximately 65% Cu, 23% O, and 12% C. The carbon content is attributed to organic compounds from the plant extract acting as capping agents. This organic layer plays an important role in colloidal stability and influences the release of Cu^2+^ ions in biological environments.

CuNPs synthesized using *Falcaria vulgaris* used for in vivo wound-healing study were described as spherical nanoparticles with an average size of 20 nm [78,82]. This intermediate particle size, together with the phytochemical coating from the plant extract, was associated with strong antimicrobial and wound-healing effects. These included low minimum inhibitory concentration (MIC) values and a significant increase in fibrocyte counts observed in vivo (see Section 4 and Section 5).

### 3.4. FTIR and Phytochemical Capping

FTIR analysis shows that polyphenols, flavonoids, and other phytochemicals are involved in both the reduction and stabilization of copper nanoparticles [43,83,84,85]. In the Krameria-based system, a strong and broad absorption band around 3422 cm^−1^ was assigned to O-H stretching vibrations of phenolic groups. Additional peaks at 2326 cm^−1^, attributed to C≡N stretching, and around 1107 cm^−1^, associated with N-H bending, were also observed. These signals indicate that cyano- and amino-containing compounds from plant extract bind to the surface of the Cu nanoparticles and act as stabilizing agents [34].

Similarly, in the synthesis using date extract, FTIR analysis showed carbonyl (C=O) and hydroxyl (O-H) bands attributed to phenolic compounds. The authors linked these groups to both the reduction of Cu^2+^ to Cu^0^ and the formation of an organic layer that stabilizes the nanoparticles [71]. In the case of CuO nanoparticles synthesized with *Morinda citrifolia* extract, FTIR spectra displayed characteristic Cu-O vibrations below 600 cm^−1^, together with bands associated with plant flavonoids and glycosides [51]. These findings support the formation of hybrid inorganic-organic nanoparticles.

Overall, these spectroscopic results indicate that the same phytochemicals responsible for antioxidant activity also influence particle size, surface charge, and interactions with biological systems. This highlights a close link between plant chemistry, nanoparticle structure, and biological performance. To better illustrate these differences, Table 1 summarizes key characterization parameters for representative green Cu and CuO nanoparticle systems discussed in both in vitro and in vivo studies.

## 4. In Vitro Biological Activities of Plant-Mediated Cu-Based Nanoparticles

**Figure 4:**
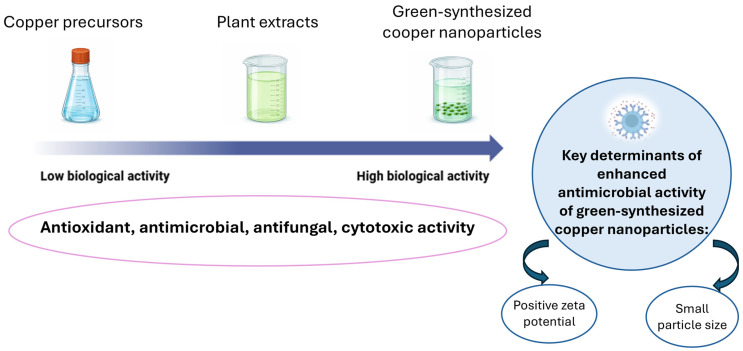
Biological activity of green-synthesized copper nanoparticles. Created in BioRender. Lungu, I. I. (2026).

In vitro studies on green-synthesized Cu and CuO nanoparticles report a wide range of biological effects, including antioxidant activity, antibacterial and antifungal action antibiofilm effects, anticancer or cytotoxic responses, and modulation of cell viability in non-malignant cells. The reported results depend strongly on factors such as nanoparticle size, oxidation state, applied dose, and the nature of the phytochemical coating (Figure 4).

### 4.1. Antioxidant Activity and Radical Scavenging

Alshammari et al. evaluated the antioxidant activity of Cu nanoparticles synthesized using *Krameria* root extract using 2,2-diphenyl-1-picrylhydrazyl radical scavenging assay (DPPH) assay. At a concentration of 200 µg/mL, CuNPs showed a radical scavenging activity of 83.81%, which was higher than that of the plant extract alone (76.02%) and copper sulfate (42.78%) at the same concentration. Increasing the CuNP concentration led to a significant rise in scavenging activity, from about 55% to over 83% [34]. These results indicate that converting copper into a nanostructured form enhances the antioxidant performance of the plant extract.

In a similar study using *Falcaria vulgaris* extract, DPPH assays showed that CuNPs exhibited antioxidant activity comparable to that of the standard antioxidant butylated hydroxytoluene (BHT) [82]. The authors attributed this effect to the combined action of the copper core and the plant-derived organic coating, which is rich in antioxidant phytochemicals and supports redox-related activity.

CuO nanoparticles synthesized using *Plumbago zeylanica* leaf extract exhibited a half maximal inhibitory concentration (IC_50_) value of 123.77 ± 1.96 µg/mL, indicating moderate radical scavenging activity. Lower IC_50_ values, corresponding to stronger antioxidant potential, were reported for CuO nanoparticles synthesized with *Bergenia ciliata* (91.2 µg/mL) and *Tribulus terrestris* (51.53 µg/mL), *Ligustrum lucidum* (63.45 µg/mL) and *Capsicum frutescens* (40 µg/mL) extracts. Among the reported systems, CuO nanoparticles synthesized using *Suaeda maritima* (28.05 µg/mL) and *Artemisia abyssinica* (5.75 µg/mL) demonstrated remarkably strong DPPH radical scavenging activity, comparable to that of conventional antioxidants [86].

Another study evaluated the antioxidant activity of CuO nanoparticles synthesized using different plant extracts compared to chemically synthesized CuO nanoparticles. The antioxidant activity was evaluated using three complementary radical scavenging assays (ABTS, DPPH and H_2_O_2_) allowing a multifaceted evaluation of antioxidant performance. In all three assays, CuO nanoparticles obtained via green synthesis showed superior antioxidant performance compared to those synthesized without plant extracts. Among the plant-mediated samples, CuO nanoparticles synthesized using *Tamarindus indica* or *Hibiscus rosa-sinensis* extracts exhibited the strongest antioxidant activity, with IC_50_ values close to those of ascorbic acid (18–22 µg/mL) against all three free radicals, highlighting their high hydrogen and electron donating capacity. Moderate antioxidant activity was observed for CuO nanoparticles derived from *Azadirachta indica*, *Murraya koenigii* and *Moringa oleifera* extracts, with IC_50_ values in the range of 25–40 µg/mL. Data suggested that plant-capped nanoparticles are more efficient in scavenging H_2_O_2_ and DPPH radicals than ABTS radicals. In contrast, chemically synthesized CuO nanoparticles without the involvement of plant extracts exhibited the highest IC_50_ values in all assays (38–54 µg/mL), demonstrating a lower antioxidant activity [87].

An in vivo study conducted in a rat model demonstrated that orally administered CuO nanoparticles (50 nm, 100 mg/kg/day for two weeks) induced marked hepatic oxidative stress, elevated liver enzymes, inflammatory response (TNF-α upregulation), and activation of apoptotic pathways, including increased caspase-3 expression. Histopathological examination confirmed liver injury and DNA fragmentation. These findings support the intrinsic pro-oxidant and hepatotoxic potential of CuO nanoparticles in biological systems [88]. In non-capped systems, CuO nanoparticles tend to promote reactive oxygen species (ROS) generation through surface redox cycling and Cu^2+^ ion release, leading to oxidative stress. However, when copper-based nanoparticles are synthesized via green routes and capped with plant-derived phytochemicals, they may shift the balance between pro-oxidant and antioxidant surface-mediated reactions. Polyphenolic compounds present on the nanoparticle surface can donate electrons, scavenge free radicals, and chelate copper ions, thereby influencing surface charge transfer processes and limiting uncontrolled ROS amplification.

### 4.2. Antibacterial and Antifungal Activity In Vitro

A substantial number of studies show that plant-derived Cu nanoparticles have strong antibacterial and antifungal activity, although the reported effects vary widely. The *Krameria*, *Morinda*, and *Falcaria* systems allow a direct quantitative comparison of this activity.

In the *Krameria* extract-based study, disc and well diffusion assays performed on Mueller–Hinton agar revealed very large inhibition zones for biosynthesized CuNPs against drug-resistant *Staphylococcus aureus* and *Escherichia coli*. At a CuNP concentration of 256 µg/mL, inhibition zones reached 43.4 mm for *E. coli* and 44.1 mm for *S. aureus*. These values were comparable to those obtained with chloramphenicol (30 µg/mL), which produced a zone of 46.2 mm and served as the positive control. In contrast, the *Krameria* root extract alone showed much weaker activity. At an MIC of 128 µg/mL, inhibition zones were only 4.7 mm for E. coli and 1.5 mm for S. aureus. This highlights the strong enhancement of antibacterial activity achieved when copper is incorporated into nanoparticles. The study demonstrated that significant antibacterial activity was achieved at concentrations as low as 32 µg/mL, indicating that effective biological activity occurs below 0.1 mg/mL [34].

The same *Krameria* CuNPs were tested against plant-pathogenic fungi (*Alternaria alternata* and *Fusarium oxysporum*). The plant extract (80 µg/mL) yielded inhibition zones between 9–11 mm against *A. alternata* and *F. oxysporum*. In contrast, CuNPs generated larger inhibition zones even at low concentrations (10–20 µg/mL) and showed statistically significant antifungal activity compared with both the plant extract and fluconazole. At high concentration (160 µg/mL), however, the difference in antifungal activity between *Krameria* CuNPs and extract was no longer significant. This effect may be due to saturation of the local environment or limitations in nanoparticle diffusion [34].

CuO nanoparticles synthesized using *Morinda citrifolia* extract showed also consistent antibacterial and antifungal activity. When tested at a dose of 25 µL of CuO nanoparticle suspension, inhibition zones were 13.6 ± 1.1 mm for *Bacillus subtilis*, 13.2 ± 0.2 mm for *Staphylococcus aureus*, and 13.1 ± 1.2 mm for *Escherichia coli* [51]. Antifungal activity was slightly higher, with inhibition zones of 13.1 ± 1.1 mm for Aspergillus flavus, 14.7 ± 0.7 mm for *A. niger*, and 16.2 ± 1.4 mm for *Penicillium frequentans*, the latter being comparable to fluconazole activity. The observation that antifungal effects matched or, in some cases, exceeded antibacterial effects can be explained by differences in fungal cell structure. Fungal spores and hyphae possess a larger surface area and a more complex, polysaccharide-rich cell wall composed primarily of chitin, β-glucans, and mannoproteins. These components provide multiple binding sites for CuO nanoparticles, facilitating enhanced adhesion, accumulation, and nanoparticle penetration [51].

In the *Falcaria* vulgaris system, antibacterial and antifungal activity was evaluatedusing minimum inhibitory concentration/minimum bactericidal concentration (MIC/MBC) and minimum inhibitory concentration/minimum fungicidal concentration (MIC/MFC) assays. Copper nanoparticles inhibited fungal growth at concentrations of 2–4 mg/mL and showed fungicidal effects at 4–8 mg/mL against *Candida albicans*, *C. glabrata*, *C. krusei* and *C. guilliermondii*. CuNPs exhibited the lowest MIC and MBC values across all tested strains, confirming their superior antibacterial potency. For Gram-negative bacteria, MIC values of CuNPs were 8 mg/mL for *Salmonella typhimurium* and *Escherichia coli*, and 4 mg/mL for *Pseudomonas aeruginosa*, while corresponding MBC values ranged from 8 to 16 mg/mL. Among Gram-positive bacteria, CuNPs showed even greater efficacy, with MIC values of 4 mg/mL for *Staphylococcus aureus* and 2 mg/mL for both *Streptococcus pneumoniae* and *Bacillus subtilis*, and MBC values between 4 and 8 mg/mL [82]. These results indicate a higher sensitivity of Gram-positive bacteria to CuNPs.

In contrast, the *F. vulgaris* extract exhibited moderate antifungal and antibacterial activity, with values generally two-fold higher than those of CuNPs. While ionic copper possesses inherent antifungal and antibacterial properties (weak antibacterial and antifungal performance of CuSO_4_), its effectiveness requires substantially higher concentrations compared to CuNPs [82]. Although these concentrations are higher than levels reported in other studies, they reflect that *Falcaria*-derived copper nanoparticles have potential as multifunctional topical agents.

The data indicate that small, metallic-rich Cu nanoparticles (5–8 nm), can produce inhibition zones comparable to antibiotics at concentrations in the tens to hundreds of µg/mL. In contrast, larger CuO nanoparticles (20–50 nm) typically generate moderate inhibition zones at similar volumetric doses [34,51,82]. These results suggest that antimicrobial potency is jointly influenced by particle size and the composition of the plant-derived surface coating. Results of antibacterial and antifungal activity are summarized in Table 2.

### 4.3. Cytotoxicity and Anticancer Effects In Vitro

Several plant-derived copper-based nanomaterials have shown selective anticancer activity while maintaining low toxicity toward non-malignant cells, although the effective concentrations vary considerably between systems.

Zangeneh et al. investigated Cu nanoparticles synthesized using *Falcaria vulgaris* extract and evaluated their effects on human umbilical vein endothelial cells (HUVECs). The authors reported high cell viability in a dose-dependent manner, with no detectable cytotoxicity even at the highest tested concentrations (1 mg/mL), suggesting good biocompatibility with normal vascular cells [82].

At the same time, these CuNPs exhibited strong cytotoxic effects against cancer cell lines. In the *Thymus fedtschenkoi* system, Dehnoee et al. reported pronounced and selective growth inhibition of multiple human lung cancer cell lines by biosynthesized CuNPs, with IC_50_ values of 173 ± 3 µg/mL for NCI-H661, 250 ± 7 µg/mL for NCI-H1975, 142 ± 5 µg/mL for NCI-H1573, and 115 ± 7 µg/mL for NCI-H1563. In contrast, no cytotoxic effect was observed toward normal human umbilical vein endothelial cells (HUVECs), even at concentrations up to 1000 µg/mL, indicating a cancer–cell selectivity [37].

A broader perspective is provided by the review of Woźniak-Budych and co-workers, which summarized multiple CuO and CuS-based theranostic nanoformulations for cancer therapy. For instance, CuS nanoparticles loaded with doxorubicin achieved nearly 68% tumour inhibition in an Ehrlich ascites carcinoma mouse model under near-infrared irradiation, highlighting the synergy between copper-based nanostructures, photothermal effects and drug delivery [78]. Although these systems are not strictly plant-mediated, they demonstrate the strong therapeutic potential of copper nano-architectures.

Overall, these studies emphasize that copper nanoparticles should not be viewed as single materials. The need to consider nanoparticles as integrated systems is broadly applicable to metal and metal-oxide nanomaterials, particularly those exhibiting redox activity and ion release (e.g., iron oxides, zinc oxide, silver nanoparticles). Copper-based nanoparticles represent a representative example of this complexity due to their dynamic oxidation-state interconversion and surface redox processes. Accordingly, factors such as plant source, particle size and phase, and surface chemistry play a decisive role in determining whether copper based nanomaterials exhibit antimicrobial, antioxidant or anticancer activity in vitro.

## 5. In Vivo Biological Effects of Plant-Mediated Cu-Based Nanoparticles

In vivo studies reported in the selected articles and related literature mainly address wound healing, anti-infective applications, anticancer effects, and plant growth promotion. The most detailed quantitative data are often obtained from hybrid CuNP systems, where plant-derived copper nanoparticles are combined with therapeutic agents such as phenytoin.

### 5.1. Cutaneous Wound Healing and Infection Control

One of the most comprehensive in vivo evaluations of copper nanoparticles synthesized using *Falcaria vulgaris* was reported by Zangeneh et al. [82]. In this study, full-thickness skin wounds were induced in rats, which were then treated for 10 days with 0.2% CuSO_4_ ointment, 0.2% *F. vulgaris* extract ointment, 3% tetracycline ointment or 0.2% CuNP ointment. The 0.2% CuNP ointment-treated group showed a statistically significant enhancement in wound healing compared to all other groups. Histological and biochemical analyses revealed accelerated wound contraction, increased angiogenesis, and elevated levels of extracellular matrix components, including hydroxyproline, hexosamine, and hexuronic acid. In parallel, CuNP treatment significantly reduced wound area and inflammatory cell infiltration, particularly neutrophils and lymphocytes, while increasing fibrocyte/fibroblast ratio reflecting progression from proliferative to remodeling phases of healing. *F. vulgaris*-derived CuNPs markedly promote tissue regeneration and modulate inflammation more effectively than the plant extract, CuSO_4_, or conventional antibiotic treatment [82].

A systematic review by Woźniak-Budych et al. further emphasized the relevance of the *Falcaria vulgaris*-based CuNP. The authors reported that ~20 nm CuNPs capped with *Falcaria* extract effectively disinfected wounds in rats and promoted the healing process [78]. Increases in fibrocyte numbers and in the fibrocyte-to-fibroblast ratio reflected enhanced extracellular matrix deposition and tissue remodeling. The same review highlights that larger CuNPs (~80 nm) accelerated blood vessel formation and wound closure in rat skin. Importantly, no changes were observed in serumalanine aminotransferase (ALT) and alkaline phosphatase (ALP), albumin, or total protein levels over 3–21 days of treatment, supporting the systemic safety of topical CuNP application [78].

Another example linking plant-mediated copper nanotechnology to wound healing involves phenytoin-loaded CuNPs synthesized using licorice extract. In a rat excisional wound model, Saddik et al. showed that this nanoformulation improved wound contraction, re-epithelialization, dense collagen deposition and reduced inflammatory cell infiltration compared with phenytoin alone, CuNPs alone, or base ointment. Notably, phenytoin-loaded CuNPs induced an approximately tenfold increase in procollagen type I expression, which is a key indicator of effective wound healing [89].

Several additional plant-derived copper nanoparticle systems, such as those based on *Allium eriophyllum* and *Artemisia annua*, have also been reported to enhance skin wound or burn healing. Overall, studies emphasize that green-synthesized CuNPs generally promote faster wound closure and improved infection control compared with conventional treatments, without causing significant systemic toxicity [22].

### 5.2. In Vivo Antimicrobial and Wound-Related Infection Outcomes

The antimicrobial MIC and MBC values reported for *Falcaria vulgaris*-derived CuNPs (see Section 4.2) were reflected in effective infection control in the in vivo wound model. Zangeneh et al. showed that wounds treated with CuNP ointment had significantly lower bacterial and fungal loads than all other treatment groups [82]. This outcome indicates not only direct antimicrobial activity but also improved barrier function, likely resulted from faster re-epithelialization. Importantly, these findings demonstrate that the nanoparticles remain effective in the complex wound environment and not only under in vitro conditions.

A review by Sandoval et al. (2022) [90] further supports the clinical relevance of copper-based wound dressings. The review analyzed several chronic wound models and concluded that copper-containing dressings consistently achieved higher wound closure rates at days 14 and 21 compared with standard treatments. Reported closure values were often in the range of 60–80%, compared with 30–50% for conventional dressings in diabetic and infected wound models. Many of these systems used Cu or CuO nanoparticles loaded-polymeric matrices, frequently incorporated plant-derived capping agents [90].

### 5.3. In Vivo Anticancer and Systemic Effects

Most in vivo anticancer studies in copper nanotechnology use chemically synthesized Cu, CuO, or CuS nanoparticles. In addition, plant-mediated systems included in this review, such as *Thymus fedtschenkoi*- and *Acroptilon repens*-derived CuNPs, are specifically developed with potential applications in translational oncology [37].

In vivo anticancer studies involving copper-based nanomaterials, including CuS nanoparticles used for photothermal therapy and copper nanoparticles functionalized with chrysin as radiosensitizers. In an Ehrlich tumour mouse model, a CuS-based theranostic system combined with near-infrared laser irradiation (808 nm, 1 W/cm^2^) achieved tumour inhibition of approximately 68% within five days of treatment [78].

Copper nanoformulations have also shown promising effects in pancreatic cancer models. Histological and apoptotic analyses of excised tumors showed a markedly higher number of apoptotic tumor-initiating cells in mice treated with CuO nanoparticles compared with untreated controls and reduced overall tumor size. These results demonstrate that CuO-NPs suppress pancreatic tumor growth by inducing apoptosis in cancer stem–like cells that are typically resistant to therapy [91].

Taken together, these findings suggest that copper nanoparticles may extend beyond topical and antimicrobial applications toward systemic anticancer therapy. However, careful toxicological evaluation, including studies on biodistribution and long-term copper accumulation, remains essential before further clinical translation.

### 5.4. Copper Nanoparticles and Plant Physiology In Vivo

Several studies examined the effects of Cu nanoparticles on plant physiology, particularly under abiotic stress conditions. For instance, biogenic CuNPs produced by *Klebsiella pneumoniae* NST2 were shown to reduce salt stress in maize by improving oxidative repair processes and lowering Na^+^ accumulation [92]. In maize exposed to drought stress, CuNP treatment also led to improved growth parameters and higher grain yield compared with untreated plants [93]. Moderate doses of CuNPs enhance plant performance under stress, whereas excessive copper, whether ionic or nanoparticulate, results in phytotoxic effects [22].

Similar effects have been reported in horticultural systems. Copper and copper oxide nanoparticles synthesized using different plant extracts have been applied to control fungal pathogens, including *Fusarium* and *Colletotrichum*. Foliar or soil application of copper nanoparticles significantly reduced the severity and incidence of *Fusarium oxysporum* infection in *Solanum lycopersicum*, with reductions in disease symptoms of more than 65% relative to controls. Nanoparticles treated plants were associated with improvements of plant vigour, including increased chlorophyll content and growth metrics [94,95]. Extract-based copper nanoparticles were also able to promote seed germination, early seedling growth, increased chlorophyll content, and detoxification enzyme activity, while also demonstrating efficient copper uptake. Examples include studies based on *Avicennia marina* and *Catha edulis* [56,57]. Taken together, these studies highlight the dual role of copper nanoparticles as both micronutrients and fungicidal agents.

Table 3 summarizes the available in vivo data and highlights plant-mediated copper systems within the broader field of copper nanomedicine.

Taken together, the in vivo findings point to three main conclusions. First, plant-mediated copper nanoparticles can significantly accelerate wound healing while effectively reducing bacterial and fungal load, as reflected by changes in biochemical markers of extracellular matrix formation and inflammation. Second, when applied at therapeutic doses in skin and tumor models, CuNPs can be designed to minimize systemic toxicity. This is supported by stable liver function parameters and the preserved viability of normal cells, such as HUVECs. Third, anticancer applications represent a growing area of interest, particularly when CuNPs are combined with photothermal or chemotherapeutic approaches.

## 6. Conclusions

From a synthesis perspective, plant-mediated routes offer a simple and versatile strategy in which reduction, stabilization, and capping occur simultaneously, without the need for toxic reagents. Across the reviewed studies, small size copper nanoparticles typically exhibited stronger antimicrobial and antioxidant effects, whereas the larger ones showed moderate bioactivity, particularly in antifungal and photocatalytic applications. FTIR and EDX analyses further demonstrate that phytochemical capping is not a passive feature but a critical determinant of colloidal stability, surface charge, and ion release behavior in biological environments.

Green-synthesized Cu and CuO nanoparticles consistently show strong antimicrobial, antioxidant, wound-healing, and selective anticancer activity. These effects are closely related to nanoparticle size, oxidation state, and the presence of phytochemical surface coatings, indicating that plant metabolites actively influence both physicochemical properties and biological behavior. The strongest in vivo evidence is currently available for topical and regenerative applications, particularly wound healing and infection control, where plant-derived copper nanoparticles achieve outcomes comparable to conventional treatments.

However, several open questions remain. First, reproducibility and standardization represent major challenges, as plant extracts vary significantly depending on species, cultivation conditions, seasonality and extraction protocols. Future efforts should focus on phytochemical profiling and quantitative standardization of extracts to enable better cross-study comparison. Second, mechanistic understanding of nanoparticle–cell interactions remains incomplete, particularly regarding cellular uptake, copper ion release kinetics, redox modulation pathways, and long-term cellular responses.

Further research should also address comprehensive toxicological evaluation, including biodistribution, chronic exposure, accumulation in organs, and potential genotoxic or immunomodulatory effects. The development of clear structure–activity relationships that correlate nanoparticle size, crystalline phase, surface chemistry, and biological effects is essential for designing copper-based nanomaterials.

Furthermore, plant-mediated copper nanoparticles could be incorporated into multifunctional systems such as polymeric wound dressings, hydrogels, targeted anticancer systems, antimicrobial coatings, electrospun nanofibrous scaffolds, bone-regenerative composites, biosensing devices or formulations aimed for plant protection in agriculture. Combining green synthesis approaches with advanced nanofabrication techniques and mechanistic biological studies will be critical for moving these materials from experimental research toward practical biomedical and technological applications.

## Figures and Tables

**Table 1:** Selected examples of green-synthesized Cu/CuO nanoparticles and key physicochemical parameters.

System/Extract	Nominal Phase	SPR Band (nm)	XRD Main Reflections	Size (TEM/SEM or DLS)	Zeta Potential	Notable Features
*Krameria* sp. root extract CuNPs [34]	Mostly Cu^0^ (fcc)	406–410 nm; optimal at 426 nm	(111), (200), (220) planes of fcc Cu	5.2–7.7 nm (TEM)	Not reported	Spherical/oval, very small size; strong SPR; intense bioactivity (antioxidant and antimicrobial)
Seedless date extract Cu/Cu_2_O NPs [71]	Cu^0^ + Cu_2_O	Single band at 576 nm	Cu peaks at 2θ = 43.27°, 50.41°, 74.17°	78 nm hydrodynamic diameter (DLS)	+41 mV	Roughly spherical; highly stable colloid; strong positive charge
*Morinda citrifolia* leaf extract CuO NPs [51]	CuO (monoclinic)	256 nm	CuO pattern; average crystallite ~29 nm	20–50 nm (TEM); 29.2 nm mean (SEM)	Not reported	Spherical, polydisperse; EDX: Cu 65%, O 23%, C 12%
*Falcaria vulgaris* leaf extract CuNPs [78,82]	Cu-based (likely Cu^0^/Cu_2_O)	Not specified	Confirmed crystalline by XRD	~20 nm (TEM/FE-SEM)	Not reported	Strong antioxidant, antifungal, antibacterial and wound-healing activity

**Table 2:** Summary of antimicrobial activities of plant-derived copper-based nanoparticles.

System/Extract and Target	Assay and Conditions	Key Quantitative Outcome	Interpretation
*Krameria* CuNPs vs. *S. aureus*, *E. coli* [34]	Well diffusion on Mueller–Hinton agar; CuNPs 1–256 µg/mL; chloramphenicol 30 µg/mL	At 256 µg/mL CuNPs: 44.1 mm (*S. aureus*), 43.4 mm (*E. coli*); chloramphenicol 46.2 mm. Extract MIC 128 µg/mL gave zones 1.5 mm (*S. aureus*), 4.7 mm (*E. coli*).	CuNPs essentially match a broad-spectrum antibiotic in zone size at sub-mg/mL concentrations; extract alone is far less active.
*Krameria* CuNPs vs. *A. alternata*, *F. oxysporum* [34]	Agar well diffusion; CuNPs 10–160 µg/mL; fluconazole control	Extract zones: 13 mm (*A. alternata*), 11 mm (*F. oxysporum*). CuNPs significantly higher zones at 10–20 µg/mL; significance vs. extract lost at 160 µg/mL.	Low-dose CuNPs outperform extract and reach activity comparable to fluconazole for *A. alternata*; concentration–response not strictly linear at high doses.
*Morinda citrifolia* CuO vs. bacteria [51]	Well diffusion; CuO NPs 15–25 µL; streptomycin control	At 25 µL: zones 13.6 ± 1.1 mm (*B. subtilis*), 13.2 ± 0.2 mm (*S. aureus*), 13.1 ± 1.2 mm (*E. coli*).	Moderate antibacterial activity; less dramatic than Krameria system, likely due to larger particle size (20–50 nm) and oxide state (CuO).
*Morinda citrifolia* CuO vs. fungi [51]	Well diffusion; CuO NPs 15–25 µL; fluconazole control	Zones: 13.1 ± 1.1 mm (*A. flavus*), 14.7 ± 0.7 mm (*A. niger*), 16.2 ± 1.4 mm (*P. frequentans*); *A. niger* zone similar to fluconazole.	CuO NPs more potent against fungi than bacteria; comparable to fluconazole for *A. niger*, indicating potential as antifungal agents.
*Falcaria vulgaris* CuNPs vs. mixed bacteria [82]	MIC/MBC; various bacterial strains	MIC 2–8 mg/mL; MBC 4–16 mg/mL.	Broad-spectrum antibacterial activity, albeit at higher mg/mL doses, likely reflecting high organic content and partially aggregated dispersions.
*Falcaria vulgaris* CuNPs vs. fungi [82]	MIC/MFC; various fungal strains	MIC 2–4 mg/mL; MFC 4–8 mg/mL.	Strong antifungal activity with similar or slightly lower MIC/MFC than for bacteria, consistent with observed wound infection control in vivo.

**Table 3:** Representative in vivo effects of copper-based nanoparticles relevant to plant-mediated systems.

System/Model	Dose/ Formulation	Duration	Key Quantitative or Semi-Quantitative Outcomes	Reference
Falcaria vulgaris CuNP ointment in rat full-thickness skin wounds	0.2% CuNP ointment vs. 0.2% CuSO_4_, 0.2% plant extract, 3% tetracycline, base, and untreated controls	10 days topical treatment	CuNPs significantly increased wound contracture, vessel count, hexosamine, hydroxyproline, hexuronic acid, fibrocytes and fibrocyte/fibroblast ratio (*p* ≤ 0.01); significantly reduced wound area, total cells, neutrophils, lymphocytes vs. all other groups; MIC 2–8 mg/mL and MBC 4–16 mg/mL against bacteria; MIC 2–4 mg/mL and MFC 4–8 mg/mL against fungi in vitro.	[82]
Topical CuNPs in rat wound models (review summary)	CuNPs ~20–80 nm embedded in hydrogels or ointments	Typically 14–21 days	Faster wound closure vs. controls (often 60–80% vs. 30–50% closure at day 14); enhanced angiogenesis; no significant changes in liver function markers (ALT, ALP, albumin, total protein).	[78]
Phenytoin-loaded CuNPs (licorice-based) in rat excisional wounds	Phenytoin-loaded CuNP ointment vs. phenytoin alone, CuNPs alone, and base	Study duration not fully specified in abstract (multi-day course)	Enhanced wound contraction and re-epithelialization; improved histological architecture; reduced inflammatory infiltration compared with all comparators; CuNPs also demonstrated antioxidant and antimicrobial effects in vitro.	[89]
Thymus-fedtschenkoi CuNPs: lung cancer and cytotoxicity	CuNPs; in vitro data: HUVEC viability intact up to 1000 µg/mL	In vitro exposure	Significant growth inhibition of NCI-H661, NCI-H1975, NCI-H1573, NCI-H1563 lung cancer lines; no cytotoxic effect on HUVECs up to 1000 µg/mL, suggesting a broad safety margin in non-malignant endothelial cells.	[37]
Copper-based photothermal nanoformulation (CuS + doxorubicin) in Ehrlich tumour-bearing mice	CuS nanoparticles combined with doxorubicin; 808 nm laser irradiation, 1.0 W/cm^2^	5 days post-injection	Tumour inhibition rate ~68% in treated mice; mechanism involves photothermal hyperthermia and chemotherapeutic release.	[78]
CuNPs in chronic wound healing (systematic review)	Various CuNP-containing dressings	Typically 14–21 days	In infected or diabetic wounds, Cu-containing dressings showed higher % wound closure at day 14 and 21 (often 60–80%) compared with conventional dressings (30–50%); improved granulation and epithelialization reported across multiple models.	[90]

## Data Availability

No new data were created or analyzed in this study. Data sharing is not applicable to this article.

## Abbreviations

The following abbreviations are used in this manuscript:

CuNPs	Copper nanoparticles
CuO NPs	Copper oxide nanoparticles
SPR	Surface Plasmon Resonance
UV–Vis	Ultraviolet–Visible spectroscopy
FTIR	Fourier Transform Infrared spectroscopy
XRD	X-ray Diffraction
SEM	Scanning Electron Microscopy
TEM	Transmission Electron Microscopy
EDX	Energy-Dispersive X-ray Spectroscopy
DLS	Dynamic Light Scattering
MIC	Minimum Inhibitory Concentration
MBC	Minimum Bactericidal Concentration
MFC	Minimum Fungicidal Concentration
IC_50_	Half Maximal Inhibitory Concentration
DPPH	2,2-Diphenyl-1-picrylhydrazyl
SAED	Selected Area Electron Diffraction
HUVECs	Human Umbilical Vein Endothelial Cells
ALT	Alanine Aminotransferase
ALP	Alkaline Phosphatase
ROS	Reactive Oxygen Species
